# Nodular Regenerative Hyperplasia of the liver in Juvenile Dermatomyositis

**DOI:** 10.1186/s12969-022-00690-x

**Published:** 2022-04-20

**Authors:** Aviya Lanis, Rita Volochayev, David E. Kleiner, Anusha Vittal, Theo Heller, Lisa G. Rider, Susan Shenoi

**Affiliations:** 1 Seattle Children’s Hospital and Research Center, 4800 Sand Point Way NE, PO Box 5371, Seattle, WA 98105 USA; 2 Environmental Autoimmunity Group, Clinical Research Branch, National Institute of Environmental Health Sciences, National Institutes of Health, Bethesda, MD USA; 3 Center for Cancer Research, National Cancer Institute, National Institutes of Health, Bethesda, MD USA; 4 Translational Hepatology Section, National Institute of Diabetes and Digestive and Kidney Diseases, National Institutes of Health, Bethesda, MD USA

**Keywords:** Nodular Regenerative Hyperplasia, Juvenile Dermatomyositis

## Abstract

**Background:**

We present two cases of Nodular Regenerative Hyperplasia (NRH) associated with Juvenile Dermatomyositis (JDM).

**Case Presentation:**

Case 1: A nine-year-old Caucasian male with refractory JDM and anti-NXP2 autoantibodies was diagnosed at age two. Over seven years, he developed arthritis, dysphagia, dysphonia, severe calcinosis, and colitis. Complications included recurrent cellulitis, infections, and hepatosplenomegaly. Multiple medications were chronically used, including prednisone, methotrexate, azathioprine, cyclophosphamide, mycophenolate mofetil, rituximab, tacrolimus, etanercept, abatacept, infliximab, and tocilizumab.

Case 2: A 19-year-old Asian female with chronically active JDM and anti-MDA5 autoantibodies was diagnosed at age 15. Symptomatology included ulcerative skin lesions, Raynaud’s phenomenon with digital ulcers, arthritis, interstitial lung disease with pulmonary hypertension, and calcinosis. Medications included chronic use of prednisone, methotrexate, abatacept, cyclophosphamide, mycophenolate mofetil, rituximab, tofacitinib, and sildenafil.

In both patients, clinical symptomatology was not suggestive of liver disease or portal hypertension, but laboratory studies revealed elevated serum transaminases with progressive thrombocytopenia and no active liver-associated infections. The first patient’s liver ultrasound showed coarse hepatic texture with mild echogenicity, splenomegaly, and portal hypertension. The second patient’s liver ultrasound was normal, but elastography indicated increased stiffness. Liver biopsy confirmed NRH in both patients.

**Conclusions:**

It is difficult to recognize NRH in JDM, as it often presents with elevated transaminases which may be mistaken for JDM muscle flare, corticosteroid-related fatty liver, or medication-related transaminitis. NRH has been associated with several medications used to treat JDM, including methotrexate, azathioprine, and cyclophosphamide, which should be discontinued if NRH develops. Providers should consider NRH in JDM patients with severe, refractory disease who have persistently elevated transaminases and persistent thrombocytopenia.

## Background

Nodular regenerative hyperplasia (NRH) is a rare vascular disorder of the liver associated with immunological or inflammatory systemic disorders and drug-related injuries that may progress to non-cirrhotic portal hypertension [1], with transformation of the hepatic parenchyma into small regenerative nodules without significant fibrosis [2]. Clinical presentation can vary from asymptomatic to a cholestatic pattern, with elevated alkaline phosphatase and bilirubin.

NRH is often under-recognized or may even be detected incidentally, and requires careful clinical consideration, with a low threshold for obtaining a diagnostic liver biopsy [1, 3]. NRH has been associated with various autoimmune, immunodeficiency, hematologic, infectious, neoplastic, and drug-related causes [2, 4]. There is a paucity of literature investigating the prevalence of NRH in autoimmune disorders, and there are no data thus far of NRH with Juvenile Dermatomyositis (JDM) [5].

Diagnosing NRH in JDM can be challenging, as elevated transaminases can be due to myositis disease activity, hepatotoxic medications or fatty liver form glucocorticoids. We present two cases of JDM with NRH and aim to raise awareness of this condition that may complicate severe, refractory JDM.

## Case Presentation

### Case 1

**Fig. 1:**
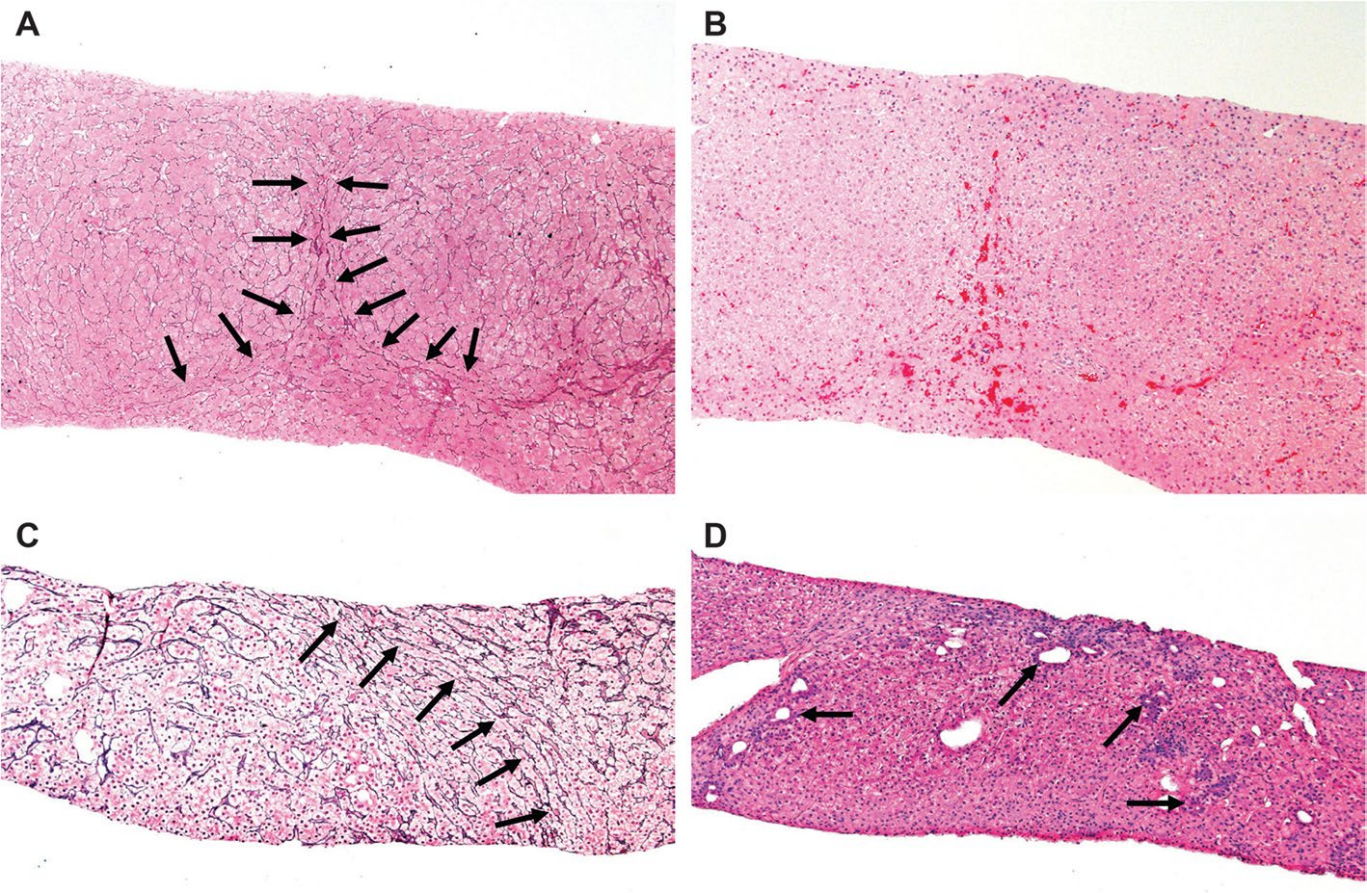
**A**: Reticulin stain showing nodularity highlighted by the variation in liver cell width from Patient 1. Plates are narrowed at the edges of the nodules (arrows) as compared to the liver cell plates in the centers of the nodules. (100x). **B**: Corresponding H&E stain of section depicted in (**A**) which shows congestion of red blood cells in sinuses between the nodules. (100x). **C**: Arrows indicate narrowed liver cell plates on this reticulin stained liver biopsy from Patient 2, consistent with nodular regenerative hyperplasia. (100x). **D**: The corresponding H&E section from Fig. 1C shows hepatocyte rosettes at the edge of the nodular area, as indicated by the arrows. (100x)

A nine-year-old Caucasian male with refractory JDM and diffuse calcinosis presented at initial diagnosis at age 2 with proximal weakness, Gottron’s papules, heliotrope rash, and arthritis. He later developed dysphagia, pharyngeal weakness requiring nasogastric tube feedings, dysphonia, and severe calcinosis with anti-NXP2 autoantibodies. The course was complicated by recurrent infections and persistent hepatosplenomegaly. He received various therapies for JDM, including chronic oral daily prednisone, intermittent pulse methylprednisolone, methotrexate, intravenous immunoglobulin (IVIG), hydroxychloroquine, azathioprine, cyclophosphamide**,** mycophenolate mofetil (MMF), rituximab, tacrolimus, etanercept**,** and abatacept, with several in combination. Three years after diagnosis, he developed diarrhea and weight loss and had patchy chronic colitis with severe ulcerations concerning for Crohn’s disease on colonoscopy, for which he received infliximab with a good response. He continued to have widespread calcinosis despite infliximab infusions. Six years into the disease, he developed persistently elevated serum transaminases (alanine aminotransferase (ALT) 41–87 U/L (normal 0–33 U/L), aspartate aminotransferase (AST) 80–100 U/L (normal 0–32 U/L)), gamma-glutamyl transferase (GGT) 72 U/L (normal 0–39 U/L), progressive thrombocytopenia 155,000–169,000 K/uL (normal 173,000–369,000 K/uL), with hemoglobin 14.2–15.3 g/dL (normal 11.5–15.5 g/dL). He did not have hematemesis, melena, ascites or other clinical features of portal hypertension. Infectious causes of transaminitis were ruled out, including HIV, hepatitis B/C, and Epstein-Barr virus. Anti-smooth muscle autoantibodies were positive, liver-kidney microsomal autoantibodies were negative, and ceruloplasmin was normal. Liver ultrasound showed coarse hepatic texture with a mildly echogenic liver, as well as splenomegaly and recanalization of the umbilical vein with collaterals in the periumbilical region, consistent with portal hypertension. Liver biopsy confirmed NRH (Fig. 1 A and B), with histopathology on reticulin stain showing nodularity with variation in liver cell plate width. Routine hematoxylin and eosin (H&E) stain showed congestion of red blood cells in the dilated sinuses between the nodules.

On diagnosis of NRH, potential hepatotoxic medications, including azathioprine and methotrexate were discontinued, given their possible association with NRH. He continues to be monitored with annual Doppler ultrasound examinations for progression of portal hypertension and development of esophageal varices, thus far showing stable findings without progression. Daily corticosteroids have been recently discontinued and he is maintained on IV tocilizumab and IVIG every two weeks, with good control of his JDM disease activity and regression of calcinosis. Most recent labs have shown mildly elevated AST to 55 IU/L, with normal serum ALT, GGT, aldolase, CK, LDH and platelets.

### Case 2

A 19-year-old Asian female with refractory JDM and anti-MDA5 autoantibodies, was initially diagnosed at age 15. Her clinical course included moderate to severe weakness, polyarthritis, Gottron’s papules with cutaneous ulcerations, and fasciitis with subcutaneous edema on thigh MRI. She also had alopecia, weight loss, fevers, and Raynaud’s phenomenon with digital ulcerations. Her course was complicated by interstitial lung disease, pulmonary hypertension, and calcinosis. Medications included long-term oral daily prednisone, intermittent pulse methylprednisolone, methotrexate, hydroxychloroquine, abatacept, cyclophosphamide, MMF, rituximab, nifedipine, and sildenafil, with several used in combination. Tofacitinib was added last to her regimen.

Despite improvement in her rash and weakness, three years following initial diagnosis she developed elevated serum transaminases (ALT 54 U/L, AST 43 U/L, GGT 89 U/L) and progressive thrombocytopenia to 154,000 K/uL. Infectious causes were ruled out and the patient did not use contraceptive pills. Anti-liver-kidney microsomal, anti-smooth muscle, and anti-mitochondrial autoantibodies were negative, and ceruloplasmin was normal. Von Willebrand factor activity was 202 U/dL, von Willebrand factor antigen 191 U/dL, and factor VIII was normal at 177 U/dL. Ultrasound of the liver, abdomen, and spleen was normal. Liver ultrasound elastography showed an elevated shear wave elastography median value of 1.63 L/s, with FibroScan 11 kPa, indicative of fibrosis. Liver biopsy showed nodular parenchyma without fibrosis with regenerative nodules bounded by atrophic liver plates on reticulin stain, consistent with NRH (Fig. 1 C and D). The patient did not have clinical history or signs concerning for liver disease or portal hypertension.

Following the diagnosis of NRH, she was referred to hepatology with monitoring for progression to portal hypertension. Intravenous cyclophosphamide was discontinued, and her JDM remains well-controlled on tofacitinib.

## Discussion

We present two patients with JDM and NRH. NRH is thought to be an adaptive reaction of hepatocytes with hyperplasia in response to vascular injury [2, 4]. Postulated causes of NRH include an antibody reaction to small hepatic vessel endothelial cells, as well as local hypercoagulation, which may predispose these patients to NRH [2, 4]. Autopsy studies of middle-aged to elderly adults suggest an overall prevalence ranging between 0.72% and 2.6% [4]. The development of NRH increases with age, while sex and ethnicity have no clear role in its development [4].

For our patients, it is unclear whether NRH developed secondary to JDM, hepatotoxic medications, or a combination of both. NRH has been associated with systemic autoimmune disorders, including inflammatory bowel disease, scleroderma, rheumatoid arthritis, systemic lupus erythematosus, polyarteritis nodosa, Behçet disease, and antiphospholipid syndrome [3, 6, 7]. An analysis of 160 patients with connective tissue disease revealed seven NRH cases in patients with lupus, systemic sclerosis, and polyarteritis nodosa. Notably, none were associated with polymyositis or dermatomyositis [8].

The association of NRH with systemic diseases may be explained by acute and chronic inflammation of intrahepatic arteries, which can lead to secondary portal venous obliteration and thrombosis of nearby portal veins [2]. Type I interferons, present in both NRH and active JDM, as well as other autoimmune diseases, may be a contributing factor and an important consideration to further understand the link between these two conditions [9, 10]. Inflammatory bowel disease may be an independent risk factor for NRH, and this should be considered in our first case who had Crohn’s disease [11]. This data, however, is based on small non-controlled studies that fall to sampling and recall bias.

NRH has also been associated with immunosuppressive drugs, including azathioprine, cyclophosphamide, methotrexate, mercaptopurine, and thioguanine, antiretroviral agents (didanosine, stavudine), birth control pills, and antineoplastic agents (oxaliplatin) [6]. Discontinuation of the drug as in our patients (methotrexate, azathioprine and cyclophosphamide) minimizes progression of hepatic disease and portal hypertension-related complications.

NRH should be considered in patients with portal hypertension without manifestations of cirrhosis, such as gynecomastia, palmar erythema, or spider nevi [4]. Many may have normal transaminases, although 11–25% of patients reported had elevated serum levels of liver enzymes [4]. GGT elevation, indicative of liver origin, is useful for NRH diagnosis in JDM, whereas elevated serum transaminases may originate from muscle or liver. NRH diagnosis is difficult in JDM, given that elevated transaminases could be attributed to myositis flare, medication toxicity (methotrexate, azathioprine, MMF, etc.), or fatty liver from chronic steroid use. Alternative hepatic pathologies to consider for liver damage include autoimmune hepatitis, chronic hepatitis, fatty liver, viral hepatitis, hepatic arteritis, malignancy, and primary biliary cholangitis [7, 12]. Of note, Case 1 had anti-smooth muscle autoantibodies and may have had a higher risk of liver comorbidities, suggesting potential need to monitor these patients more closely for NRH.

NRH is often under-recognized and missed on initial ultrasound, which may not demonstrate regenerative nodules due to small size or iso-echogenicity (as was seen in the second patient). Hepatic CT and MRI are unreliable due to poor sensitivity and specificity [4]. When available, liver ultrasound elastography and FibroScan can be considered to look for liver fibrosis, which is often present. Liver biopsy is the gold standard for NRH diagnosis and requires a high index of suspicion, with review by an experienced pathologist to avoid missing the diagnosis in the early stage. Clinicians should notify the pathologist with suspected non-cirrhotic portal hypertension and request reticulin staining of liver biopsies for NRH. Microscopically, NRH presents as diffuse fine nodularity of the liver (1–3 mm diameter), that appear paler than surrounding normal hepatic tissue. Reticulin and Masson trichrome staining reveals no or limited perisinusoidal and portal fibrosis [4].

Approximately 50% of NRH cases are complicated by clinically overt portal hypertension (as noted in Case 1) [4], which may progress to esophageal varices, splenomegaly, and thrombocytopenia [2]. Annual hepatologist evaluations to monitor progression and complications of NRH is recommended, as is being done with both these patients. Case 2 has well-managed JDM with stable NRH and no signs or symptoms of portal hypertension. Portal hypertension and varices have not progressed with Case 1, once JDM disease activity was better controlled with tocilizumab therapy, suggesting JDM disease control may be an influential factor in the progression of NRH.

Patients should also be monitored for porto-pulmonary hypertension [13] or hepatopulmonary syndrome, as described in immune-deficiency disorders such as common-variable immunodeficiency. Often, development of hepatopulmonary syndrome leads to liver transplantation, although NRH may recur after transplant [14].

## Conclusions

NRH should be considered in patients with severe, refractory JDM with unexplained persistently elevated transaminases, GGT and thrombocytopenia. This facilitates discontinuing hepatotoxic medications and surveillance for complications.

## Data Availability

Data sharing is not applicable to this article as no datasets were generated or analyzed during the current study.

## Abbreviations

AST: Aspartate transaminase
ALT: Alanine aminotransferase
ALP: Alkaline phosphatase
CK: Creatinine kinase
CT: Computed Tomography
GGT: Gamma-glutamyl transferase
H&E: Hematoxylin and eosin
IVIG: Intravenous Immunoglobulin
JDM: Juvenile Dermatomyositis
LDH: Lactate dehydrogenase
MMF: Mycophenolate mofetil
MRI: Magnetic resonance imaging
NRH: Nodular Regenerative Hyperplasia
